# Hyperspectral Imaging for Evaluating Impact Damage to Mango According to Changes in Quality Attributes

**DOI:** 10.3390/s18113920

**Published:** 2018-11-14

**Authors:** Duohua Xu, Huaiwen Wang, Hongwei Ji, Xiaochuan Zhang, Yanan Wang, Zhe Zhang, Hongfei Zheng

**Affiliations:** 1 Tianjin Key Laboratory of Refrigeration Technology, Tianjin University of Commerce, Tianjin 300134, China; xudh_1993@163.com (D.X.); jhwtj@126.com (H.J.); zhxc@tjcu.edu.cn (X.Z.); zhangzhe@tjcu.edu.cn (Z.Z.); zhenghf1544@163.com (H.Z.); 2 School of Mechanical Engineering, Tianjin University of Commerce, Tianjin 300134, China; 3 School of Engineering, Deakin University, Waurn Ponds campus, Geelong, Victoria 3216, Australia; yanan.wang@deakin.edu.au

**Keywords:** impact damage, hyperspectral imaging, partial least squares regression, quality attributes, mango

## Abstract

Evaluation of impact damage to mango (*Mangifera indica* Linn) as a result of dropping from three different heights, namely, 0.5, 1.0 and 1.5 m, was conducted by hyperspectral imaging (HSI). Reflectance spectra in the 900–1700 nm region were used to develop prediction models for pulp firmness (PF), total soluble solids (TSS), titratable acidity (TA) and chroma (∆b*) by a partial least squares (PLS) regression algorithm. The results showed that the changes in the mangoes’ quality attributes, which were also reflected in the spectra, had a strong relationship with dropping height. The best predictive performance measured by coefficient of determination (*R*^2^) and root mean square errors of prediction (RMSEP) values were: 0.84 and 31.6 g for PF, 0.9 and 0.49 ^o^Brix for TSS, 0.65 and 0.1% for TA, 0.94 and 0.96 for chroma, respectively. Classification of the degree of impact damage to mango achieved an accuracy of more than 77.8% according to ripening index (RPI). The results show the potential of HSI to evaluate impact damage to mango by combining with changes in quality attributes.

## 1. Introduction

As one of the most important fruits, mango production occupies a leading position in tropical and subtropical regions in the world [1]. However, problems influencing mango quality have limited the consumption of this fruit, among which mechanical damage is a key factor that cannot be underestimated [2]. Due to the susceptibility to mechanical damage during harvesting, packaging and transport, a certain degree of decline in mango quality will occur. Therefore, it is crucial to develop rapid, non-destructive and reliable methods to evaluate mechanical damage to mango.

With regard to different kinds of mechanical damages, impact damage is the most severe and most likely type to occur. Once damaged, fruits will react with significant physiological responses in terms of ethylene production, respiration and transpiration [3,4], which may accelerate ripening of fruits during storage [5]. Numerous studies have attempted to assessed the degree of damage by detecting surface damage or by means of mechanical parameters, for example, impact energy, absorbed energy and peak force, etc. [6,7,8,9,10]. In addition, some studies have assessed the maturity according to impact responses together with statistical analysis characterized by fruits’ firmness, moisture content and so forth [11,12,13]. However, the accuracy and robustness of the models used in these studies need to be improved.

HSI technology has been widely applied to food science, in areas such as the analysis of green coffee beans [14], food grains [15] as well as quality evaluation of agro-food products [16,17], which is largely based on its distinct advantages of being a nondestructive, rapid and accurate analysis method. Moreover, HSI has been used to assess the mechanical damage of fruits [18,19,20]. However, these studies mainly focused on differentiating between damaged and undamaged fruits based on various imaging processing means and quantification of damage is far from being achieved [5,21,22,23]. Furthermore, previous research has demonstrated the potential of near infrared (NIR) spectroscopy to nondestructively determine quality attributes in mango [24,25,26,27,28] but rarely by means of HSI. Though it is possible to detect mango damages by using visible cameras or other visible technologies, what they focused on is predicting of mangoes’ quality parameters rather than attempting to connect these with the degree of damage to fruits. Thus, based on existing studies on mango damage by other various approaches or visible technologies, evaluating the degree of impact damage by NIR-HSI and correlating it with changes in quality attributes of mango can be considered as a promising research direction.

In this paper, evaluation of impact damage to mango caused by dropping the fruits from three different heights was conducted by HSI. In the damaged area of the mango sample (the region of interest), the changes in spectra of damaged mango vary and are positively correlated with the degree of impact damage. Thus, it is reasonable to characterize the degree of the impact damage by changes in the quality attributes of mango. The aim is to explore the feasibility of assessing the effect of impact damage on quality attributes of mango by HSI, and in turn, to evaluate the degree of impact damage according to changes in quality attributes.

## 2. Materials and Methods

### 2.1. Experimental Procedure

**Figure 1:**
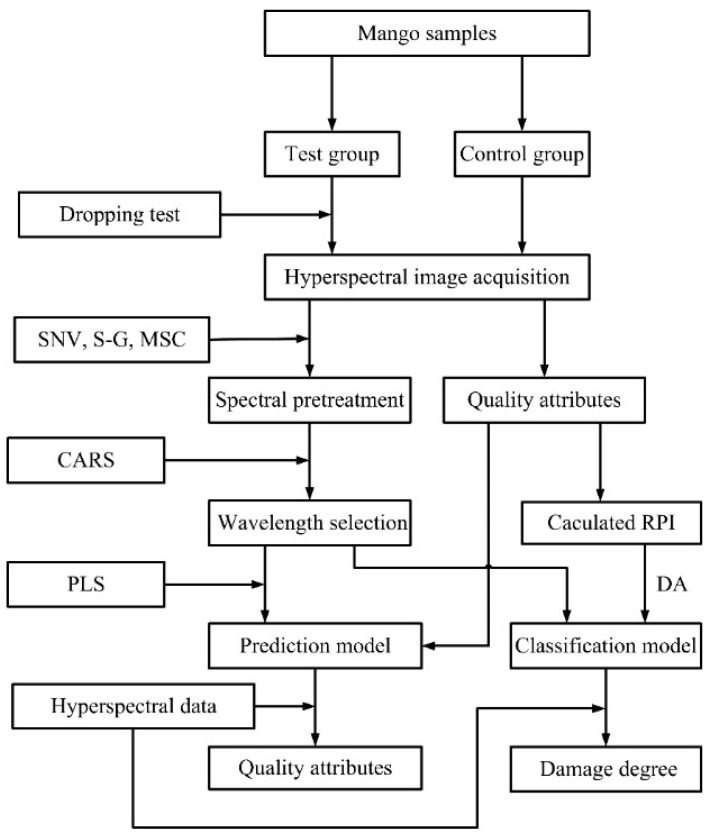
Flow chart of the process of evaluating mechanical damage to mango by HSI.

The entire experimental procedure is sketched in Figure 1. The specific steps to evaluate mechanical damage to mango by HSI are as follows:

(1) Obtain hyperspectral data and quality attributes of damaged and undamaged mangoes.
(2) Develop prediction models for the correlations between spectral data and quality parameters of tested mango samples for non-destructive quality prediction of individual mango.
(3) Develop a classification model between spectral data and RPI values derived from quality parameters for the prediction of degree of impact damage to mango.

### 2.2. Preparation of Mango Samples

Unripe mangoes (240) were selected from the same batch, purchased from a local market in Tianjin. Before experiments, the mangoes’ surfaces were cleaned and numbered. Impact damage was induced in the equatorial zone of fruits using a drop test machine (PD-315A, Suzhou New District Dongling Vibration Testing Instrument Co., Ltd., Suzhou, China). The maximum load of the machine is 100 kg and drop height is 0.3–1.5 m. Each bruised area was marked so as to be able to identify the damaged area. All mango samples were grouped into three groups for three days’ experiments and each group was grouped into two subgroups. One subgroup with 20 mangoes were free from damage. Another subgroup with 60 mangoes were dropped onto a steel plate from three different heights (0.5, 1.0 and 1.5 m, respectively). In the dropping experiments, 20 mangoes were dropped from the same dropping height. Immediately after the mangoes were dropped, the samples were stored under conditions of darkness at a room temperature about 20 °C and relative humidity of 42% for further data acquisition.

### 2.3. Hyperspectral Image Acquisition

Hyperspectral images of the mangoes were acquired 24 h after the damage was caused and then another two times every other day (i.e., day1, day3 and day5) using a hyperspectral imaging acquisition system (Imspector N17, Spectral Imaging Ltd., Oulu, Finland). A line-scanning CCD hyperspectral camera, a translation stage and illumination units (four halogen lamps, 35 W) are included in HSI system. The drive software is SpecView. The reflectance mode (log 1/R) was used to carry out spectral acquisition within the spectral range 900–1700 nm. A total of 256 spectral bands were recorded by the system. Thus, the spectral resolution was approximately 3 nm. Specific parameters were set as follows: exposure time was 20 ms, the speed of translation stage was 14 mm/s, the distance between the lens and samples was 420 mm.

Before collecting hyperspectral images of samples, black and white calibration was performed under the same test condition as samples’ hyperspectral image acquisition in order to reduce the effect of dark current of camera and changes of light intensity on the image signal.

First the white calibration image (*W*) was obtained by scanning the polytetrafluoroethylene (PTFE) standard calibration whiteboard. Then the black calibration image (*B*) was got by covering the lens cap. Finally, the calibrated image (*I*) was calculated by the original hyperspectral image (*I*_0_) following Equation (1):

(1) $$I=\frac{I_{0}-B}{W-B}\times 100\;$$

After collecting the hyperspectral images of the mango samples, the damaged areas of samples in the test group and any area of samples in the control group were selected as the region of interest (ROI) to extract the average spectra. The original spectra were the set of the average spectra extracted from all samples.

### 2.4. Determination of Quality Attributes

#### 2.4.1. Pulp Firmness

A texture analyzer (TA.XT plus, Stable Micro Systems Ltd., Godalming, UK) was used to perform puncture tests using a Φ = 2 mm diameter stainless needle probe at a speed of 0.5 mm/s. The compression depth was set as 10 mm. The average force (g) from 2 to 3 s during the penetration process was calculated as PF (g). Before tests, the peel of the mango sample was removed and puncture tests were conducted in the damaged area.

#### 2.4.2. Total Soluble Solids

A portable refractometer (PAL-1, Atago, Tokyo, Japan) within range from 0 to 32 ^o^Brix was used to measure TSS of 1 mL mango juice and the results were expressed as ^o^Brix. It should be noted that the mango juice was extracted from the mango’s damaged areas.

#### 2.4.3. Titratable Acidity

A traditional titration method was used to measure TA in mango samples. Phenolphthalein was used as the titration indicator and NaOH solution was the titrant. Mango juice (10 g) was extracted from each mango sample and diluted with distilled water (100 mL). The diluted sample was subjected to water bath heating for 30 min at a temperature of 80 °C. After cooling, the volume was adjusted to 250 mL by adding distilled water again in order to conduct filtering step. Then a portion of the filtrate no filtration step mentioned so where does this come from? (50 mL) was obtained and 1–2 drops of phenolphthalein were added. Finally, the NaOH solution was slowly added until neutralization occurred. The volume of consumed NaOH solution was recorded as *V* (mL). The results were expressed as mass percentage of citric acid (%) calculated as:

(2) $$TA(\%)=\frac{C\times V\times K}{M\times \frac{V_{1}}{V_{0}}}\times 100\;$$

where *C* stands for the concentration of NaOH solution, i.e., 0.1 mol/L. *V* stands for the volume of NaOH solution consumed during the titration. *M* stands for the quantity of mango juice. *V*_1_ stands for the volume of the filtrate and *V*_0_ stands for the total volume of the diluted solution. *K* stands for coefficient converted to the main acid, that is, grams of the main acid that are equivalent to 1 millimole of NaOH. Since the acids in mango are dominated by citric acid, the *K* value is 0.064.

#### 2.4.4. Flesh Color

Flesh color was determined on each mango piece cut from a damaged area (test group) or an undamaged area (control group) of individual mangoes with a colorimeter (Ultrascan Pro, Hunter Associates Laboratory, Inc., Reston, VA, USA). The color value is represented by chroma (∆b*) of the Commission Internationale de L’Eclairage (CIE) color space. Positive values of ∆b* indicate that the color of the sample is more yellowish than the standard color, and greenish otherwise.

#### 2.4.5. Ripening Index

In all of the above measurements, the selected measurement area should be as consistent as possible with ROI selected during the process of extracting the mean spectra. Subsequently, to describe the ripeness of mango samples comprehensively and to classify the degree of damage, a ripening index (RPI) was calculated according to [29]:

(3) $$\mathrm{RPI}=\ln\frac{100\times \mathrm{Firmness}\times TA}{TSS}\;$$

It is worth noticing that such characterization of impact damage by means of RPI is an indirect way, which is also our original intention for introducing the ripening index. Thus, the determination of mango ripeness is only one step of this process and not our final goal.

## 3. Spectral Analysis

### 3.1. Spectral Preprocessing Methods

Spectral preprocessing is necessary to improve the accuracy of prediction model. Savitzky-Golay smoothing (S-G), standard normal variate (SNV) transformation and multiplicative scatter correction (MSC) were evaluated in this study. S-G smoothing method uses the least squares fitting coefficient to establish the filtering function, which performs polynomial least squares fitting on the spectral data in the moving window instead of simple average. MSC aims to obtain a more “ideal” spectrum by correcting the scattering of each spectrum. This algorithm assumes that each spectrum should be linearly related to the “ideal” spectrum. Like MSC, SNV can also be used to correct the spectral errors caused by scattering between samples, which carries on the standard normalization to each original spectral data and avoids the need to obtain the “ideal” spectrum.

### 3.2. Modeling Procedure

In order to improve the efficiency of prediction models, competitive adaptive reweighted sampling (CARS) was used for selecting important wavelengths. Prediction models were developed using partial least squares (PLS) regression based on both full spectral range (900–1700 nm) and selected key wavelengths variables. The principles and procedures of corresponding algorithms are described as follows.

## 4. CARS

This algorithm combined with the PLS algorithm aims to eliminate variables with smaller absolute weight of regression coefficients in PLS regression model and to give priority to variables with larger absolute weight of regression coefficients. According to the cross validation, the subset of variables with the least error of the root mean square (RMSECV) are chosen and the variables contained in this subset are the combination of the optimal characteristic wavelength variables. The main steps in CARS for key wavelengths selection have been described in detail in [30].

### 4.1. PLS Modeling

The PLS algorithm combines factor analysis and regression analysis. The main procedures are described as follows:

First, we decompose the spectral matrix *X* and quality attributes matrix *Y*:

(4) X=TP+E 

(5) Y=UQ+F 

where *T* and *P* are the scoring matrix and the load matrix of *X*, respectively; *U* and *Q* are the score matrix and the load matrix of *Y*, respectively; *E* and *F* are the error matrices introduced when the model is fitted with *X* and *Y.*

Then, to establish a linear regression relationship according *T* and *U*:

(6) U=TB 

where *B* is the regression coefficient matrix. Finally, we predict the parameters of unknown samples to be measured:

(7) $$Y_{un}=T_{un}BQ+F\;$$

That is to say, the score matrix *T_un_* of the unknown sample *X_un_* is obtained from the *P* matrix, and then *Y_un_* is calculated from the formula.

During the analysis process, three-fifths of the samples were grouped into modeling sets (calibration sets) and the remaining samples were included in prediction sets (validation sets). That is, for each group, there were 12 samples in the calibration sets and eight samples in the validation sets. The predictive performance of the developed models was characterized by the coefficient of determination of prediction (*R_P_*^2^) and root mean square error of the predicted values (RMSEP). High *R_P_*^2^ combined with low RMSEP indicates satisfactory predictive performance. Specifically, the leave-one-out method was used in the cross-validation process during PLS modeling.

### 4.2. Classification of Damage Degree

RPI, where lower values mean increased ripening, was used to define fruit ripeness as described previously [29,31,32]. The investigation results indicate that the RPI values of damaged mangos are significantly different from these of undamaged mangoes under the same conditions, such as the dropping height, temperature used for storage and so on. Thus, RPI values could be used to characterize the degree of impact damage. Mango samples were classified into three groups according RPI values. Mangoes which had an absolute RPI value of greater than 7.0 were considered slightly damaged, whereas mangoes having RPI of less than 5.0 were classified as seriously damaged. Mangoes with RPI of 5.0–7.0 were defined as moderately damaged. Discriminant analysis (DA) was applied to spectral variables selected by CARS to classify mangoes according to RPI. ‘Fisher’s’ and ‘Unstandardized’ methods were selected to give the coefficients of the discriminant function. Three fourths of the samples were defined as calibration sets (training sets) for development for the classification model and the remaining samples were considered as validation sets (testing sets) to test the efficiency of classification.

## 5. Results and Discussion

### 5.1. Quality Characteristics after Damage

**Figure 2:**
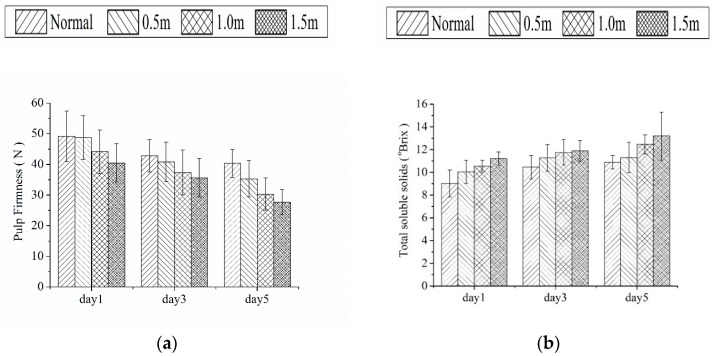

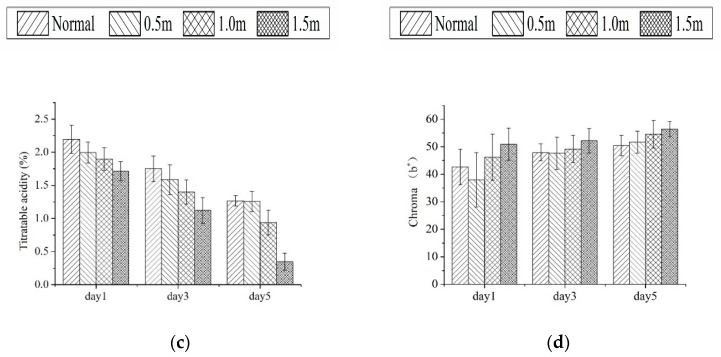
Changes in quality attributes of mango samples both in normal and damaged stages (**a**) pulp firmness, (**b**) total soluble solids (TSS), (**c**) titratable acidity (TA) and (**d**) chroma (∆b*).

The trend of quality attributes of the tested mangoes with changes of dropping height and test dates, is shown in Figure 2.

From Figure 2, the following conclusions may be reached: (1) For the same dropping height, values of PF and TA tended to decrease, whereas TSS and chroma increased over time throughout the observation process, which is consistent with typical ripening characteristics. More importantly, samples that are subjected to impact damage have a more pronounced performance as discussed above; (2) For different dropping heights, during observation in the same day, the higher dropping height is, the more pronounced performance will be. Additionally, the samples used in this study showed variability for the quality parameters due to the diversity of physiological development of the fruits themselves.

The related statistical parameters of the samples’ quality attributes are listed in Table 1. It can be found from Table 1 that the differences are statistically significant. In particular, it is important to note that the lower dropping height is, the larger the RPI values will be in the same day. Furthermore, the RPI values tended to decrease for the same dropping height over time. The above results indicate that it is reasonable to classify the degree of impact damage according RPI values.

### 5.2. Spectral Analysis

Generally, absorption peaks appear in the spectra of fruits because of the presence of certain particular chemical bonds [33], involving O–H, C–H and N–H bonds existing in fruit constituents such as water, carbohydrates and organic acids [34,35,36]. In general, the water absorption bands, which dominate the NIR spectra of fruits and vegetables, are normally wide and centered at approximately 970, 1450, 1950 and 2250 nm [37,38]. The absorption bands of starch and sugars are normally found at 1190 nm [39], where most of them are overlapping with the broad water bands around the of 970 and 1450 nm region [2]. Monomeric organic acids normally show bands related to the O–H group from the first, second and third overtone at about 1445, 1000 and 800 nm, respectively [40].

**Figure 3:**
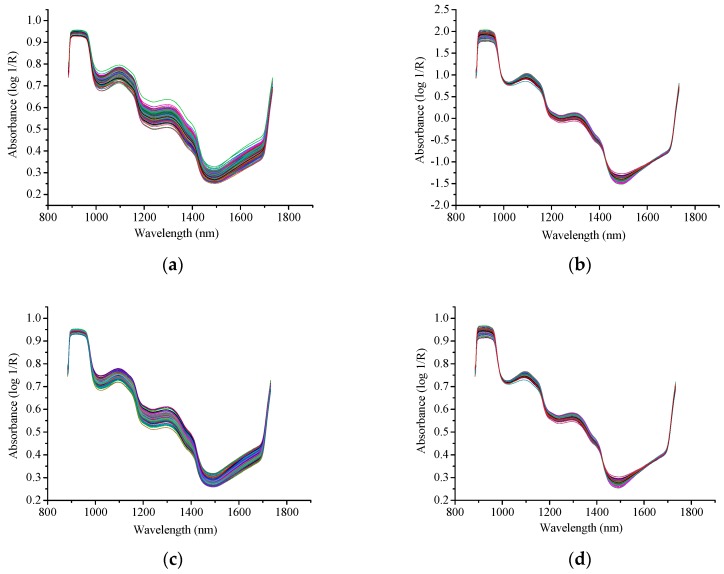
Spectra of all mango samples in full spectral range. (**a**) Raw spectra; (**b**) Spectra preprocessed by SNV; (**c**) Spectra preprocessed by S-G; (**d**) Spectra preprocessed by MSC.

Before further analysis, all the raw absorbance spectra (after reflectance correction) of the 240 mango samples were subjected to preprocessing. Raw spectra and corrected spectra by different preprocessing methods are shown in Figure 3, where it can be seen that the performance of S-G is the worst compared to the other two methods.

**Figure 4:**
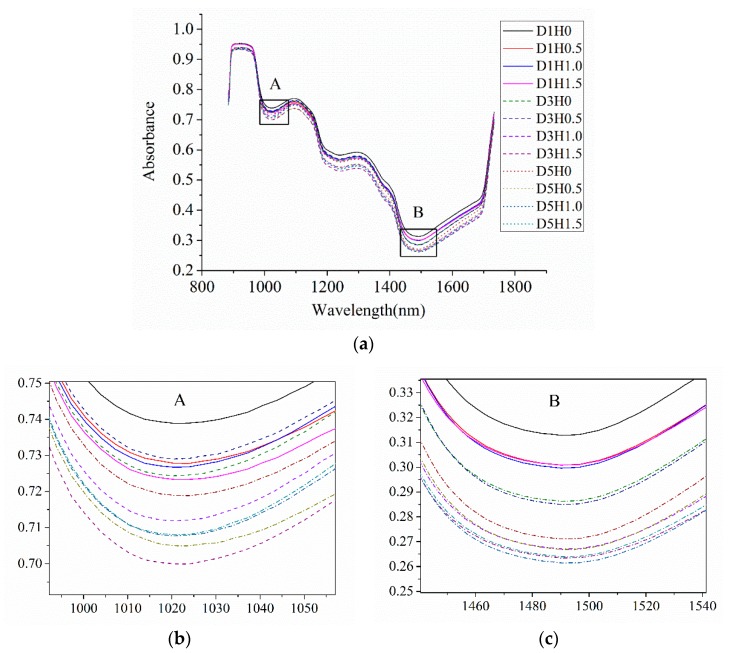
Mean spectra of mango samples (damaged and undamaged) for three days at three different dropping heights. (**a**) overall mean spectra; (**b**) enlarged picture near the wavelength 980 nm; (**c**) enlarged picture near the wavelength 1500 nm.

The two characteristic water absorption bands at 970 and 1450 nm are still obviously observed, although the distance from the characteristic peak is somewhat far. Mean spectra obtained from different dropping heights for three days are illustrated in Figure 4. The legend “D*m*H*n*” means mango samples dropped from height *n* with hyperspectral images collected on day *m*. For example, D1H0.5 means the mango samples that dropped from the height of 0.5 m and hyperspectral images collected on the first day. It can be seen that the IR absorptions of damaged mangoes’ spectra are lower than that of undamaged mangoes. The higher the dropping height is, the lower the IR absorption will be. Certainly, the deviation of spectral bands may exist within certain spectral range, which may be ascribed to the changes of optical properties influenced by the alteration of physicochemical properties [32,41].

### 5.3. Modeling Results

After modeling by spectral data preprocessed by SNV and MSC respectively, it is seen that SNV can improve the prediction accuracy of the model in a certain extent. Thus, SNV is the best preprocessing method among these three approaches. Therefore, the following prediction models for all quality parameters were built using spectra corrected by SNV.

**Figure 5:**
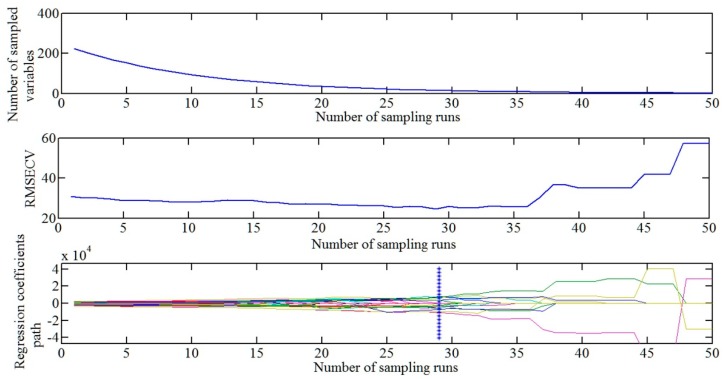
Changes of the number of sampled variables, RMSECV and regression coefficients path with the increase in the number of sampling.

After spectra preprocessing, competitive adaptive reweighted sampling (CARS) was used for selecting important wavelengths. After 50 times of sampling through competitive adaptive reweighting algorithm, 50 subsets of variables are obtained. It can be seen from Figure 5 that when the number of sampling is up to 29, the cross-validation root mean square error of the variable subset is the smallest. Corresponding optimal characteristic wavelengths were obtained, namely 1047, 1070, 1090, 1110, 1296, 1373, 1380, 1383, 1386, 1393, 1396, 1416, 1463, 1470, 1476, 1520, 1540, 1583, 1586, 1636, 1689 and 1696 nm, etc.

The prediction models were built using two different strategies: (1) using all variables on the spectral range of 900–1700 nm in order to make preliminary observation of the relationship between wavelengths and quality attributes and (2) using only the variables selected by CARS. The modeling results show that there is strong relationship between wavelengths and changes in quality attributes and the latter method is better than the former one both in efficiency and accuracy to some degree. The following analysis are based on predictions obtained by the latter method.

**Figure 6:**
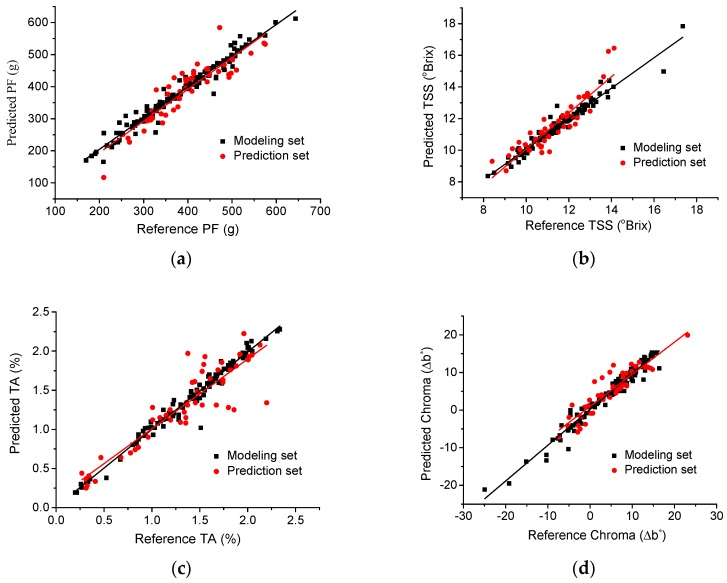
Scatter plots of predicted and reference quality attributes from both modeling sets and prediction sets for (**a**) pulp firmness (PF); (**b**) total soluble solids (TSS); (**c**) titratable acidity (TA) and (**d**) chroma (∆b*).

The scatter plots of modeling results for mangoes’ quality attributes by PLS are shown in Figure 6. It is worth noticing that Figure 6 gives the modeling results in case of calibration sets and cross-validation sets, taking all groups into account. In case of prediction sets for each group, Table 2 shows the regression statistics of PLS to predict PF, TSS, TA and flesh color from impact tests.

On the whole, the modeling results achieved by PLS to predict mangoes’ quality attributes are satisfactory. The best results of *R_P_*^2^ and RMSEP values were: 0.84 and 31.6 g for PF, 0.9 and 0.49 ^o^Brix for TSS, 0.65 and 0.1% for TA, 0.94 and 0.96 for chroma, respectively. Nonetheless, taking all groups into account, prediction of TSS and PF achieved better and more robust results.

Predicted versus reference plots for predicting PF are shown in Figure 6a, among which the highest *R_P_*^2^ is 0.84 and with the RMSEP of 31.6 g. The results were slightly satisfactory and may be a result of the lower number of samples used for developing models. The performance of model could also be improved by attempting to change methods of selecting characteristic wavelengths in future work.

For TSS, the predicted versus reference plots are shown in Figure 6b. The highest *R*^2^ was up to 0.9 with RMSEP of 0.49 ^o^Brix as shown in Table 2. Related studies have also obtained high accuracy for soluble solids (SS) determination in mango and the highest *R_P_*^2^ was up to 0.93 with RMSEP of 1.223 ^o^Brix by near-infrared spectrometry [42]. Thus, HSI for TSS prediction in this study achieved good results both in accuracy and robustness compared with previous reports.

Previous studies have demonstrated that the prediction of acidity-related parameters of fruits is challenging, especially when the TA value of mango fruit is less than 0.2%. Although the best prediction performance were obtained with *R_P_*^2^ of 0.5 and RMSEP of 0.17% [2], the robustness of calibration model needs to be further improved. As observed in Figure 6c in current study, the highest *R_P_*^2^ was up to 0.65 combined with RMSEP of 0.1%, which achieved satisfactory results.

With respect to color parameter, the application of it to assess the maturity of fruit is relatively less, especially in terms of mechanical damage. Take chroma (∆b*) as an example, where the larger values are, the higher ripeness will be. The potential of PLS model to predict chroma is promising according to Figure 6d and the data listed in Table 2.

Likewise, the prediction process was conducted by calculated RPI values, whereas modeling performance turned out not as we expected and negative values of *R*^2^ appeared. On the one hand, RPI itself is an indirect parameter and cannot be better associated with spectra data. On the other hand, the physiological changes of damaged mangoes are too complex to characterize well the damage mechanism simply by RPI. Nonetheless, from the statistical data listed in Table 1, it is obvious that the trend of RPI values is positively correlated with the degree of dropping height. That is to say, the higher dropping height is, the higher ability to accelerate the maturity of mango, which means that the more serious damage to mango will be. In other words, it is reasonable to evaluate the degree of impact damage according to RPI values.

### 5.4. Classification

The capability of hyperspectral spectra to categorize the degree of impact damage to mango was evaluated using discriminant analysis (DA). The classification results showed that 83.3% of fruits in the training set were properly categorized into the slight damaged group, and 100% of fruits in the test set were correctly classified. As shown in Table 3, 65.3% of the fruits are classified into moderate degree of impact damage while a more accurate result of 77.8% was achieved in the test sets. It is also worth mentioning is that the serious degree of impact damage classification results both in the training set and test set an accuracy of 100% was obtained. The slight and moderate damage groups were completely distinguished from the serious damage group, whereas misclassification was observed between the slight and moderate groups, which most likely due to the similarities in their spectra. With these classification performances, models showed a high potential for classifying mangos according to RPI.

## 6. Conclusions

Impact damage to mango was evaluated by HSI according to changes in quality attributes. Prediction models by PLS within the spectral region of 900–1700 nm were developed for quality attributes. The predictive performance measured by coefficient of determination (*R*^2^) and root mean square errors of prediction (RMSEP) values was satisfactory. In addition, classification of degree of impact damage to mango was successfully conducted by DA with an accuracy of more than 77.8% according to RPI values, which also shows the capability of HSI to categorize the degree of impact damage to mango.

The present studies indicate that HSI could be used as a rapid, nondestructive and reliable tool for quality control of postharvest products, which is expected to help develop industrialized automatic inspection and grading of products. Despite this, the evaluation of damage by HSI is still in the aspect of qualitative detection. Thus, our future work aims to improve the accuracy and robustness of models as well as quantify the mechanical damage to fruits by HSI.

## Figures and Tables

**Table 1:** The statistical parameters of measured quality attribute data of all mango samples.

Parameter	Drop Height	Day1		Day3		Day5	
	(m)	Mean	SD	Mean	SD	Mean	SD
	0.5	48.79	7.09	40.85	6.42	35.31	5.94
Firmness (N)	1.0	44.16	7.11	37.4	7.31	30.32	5.28
	1.5	40.51	6.19	35.66	6.24	27.72	4.08
	0.5	10.05	1.01	11.27	1.16	11.31	1.33
TSS (^o^Brix)	1.0	10.54	0.51	11.76	1.12	12.46	0.85
	1.5	11.19	0.6	11.87	0.91	13.2	2.11
	0.5	1.99	0.16	1.59	0.42	1.26	0.15
TA (%)	1.0	1.89	0.17	1.40	0.11	0.94	0.19
	1.5	1.71	0.14	1.12	0.18	0.35	0.13
	0.5	38.03	9.86	47.66	5.86	51.71	4.0
Chroma(b*)	1.0	46.25	8.38	49.22	4.95	54.52	5.01
	1.5	50.92	5.83	52.24	4.45	56.51	2.79
	0.5	6.88	0.24	6.4	0.38	6.13	0.17
RPI	1.0	6.76	0.23	6.08	0.2	5.3	0.58
	1.5	6.29	0.02	5.73	0.55	3.78	0.69

**Table 2:** Modeling results by PLS regression to predict PF, TSS, TA and flesh color from impact tests.

Parameter	Drop Height	Day1		Day3		Day5	
	(m)	*R_P_* ^2^	RMSEP	*R_P_* ^2^	RMSEP	*R_P_* ^2^	RMSEP
	0.5	0.45	4.29	0.79	3.57	0.84	3.16
Firmness (N)	1	0.79	1.86	0.67	3.52	0.74	2.17
	1.5	0.8	2.46	0.57	3.53	0.43	4.57
	0.5	0.71	0.45	0.72	0.54	0.73	0.77
TSS (^o^Brix)	1	0.66	0.21	0.89	0.36	0.9	0.49
	1.5	0.88	0.16	0.87	0.38	0.64	1.28
	0.5	-0.07	0.13	0.65	0.1	-6.92	0.28
TA (%)	1	0.58	0.06	0.22	0.16	0.46	0.11
	1.5	0.48	0.12	0.58	0.14	0.62	0.07
	0.5	0.57	3.63	0.76	1.69	0.88	1.6
Chroma (∆b*)	1	0.63	4.46	0.91	1.89	0.94	0.96
	1.5	0.88	1.72	0.8	2.91	0.82	1.15

**Table 3:** Classification results for damage degree of mango performing discriminate analysis (DA) on spectral data according to RPI.

Actual Group (%)		Classified Group (%)			Total (%)
		Slight	Moderate	Serious	
Training set	Slight	83.3	16.7	0	100
	Moderate	27.6	65.3	7.1	100
	Serious	0	0	100	100
Testing set	Slight	100	0	0	100
	Moderate	20.7	77.8	1.5	100
	Serious	0	0	100	100
